# National Survey of Narrative Assessment in LCME-accredited Anatomy Laboratories

**DOI:** 10.1007/s40670-026-02861-x

**Published:** 2026-08-25

**Authors:** Helen Kaiser, Christian Cormas

**Affiliations:** Biomedical Sciences Departmnet, University of South Carolina School of Medicine Greenville, 607 Grove Rd, Greenville, SC 29605 USA

**Keywords:** Narrative assessment, Anatomy education, Feedback as coaching, LCME accreditation, medical education survey

## Abstract

Narrative assessment is a key component of competency-based medical education, providing qualitative information that supports learner development and professional growth. LCME Element 9.5 requires narrative assessment whenever teacher–student interaction permits, yet its implementation in anatomy laboratories remains poorly characterized. This descriptive, cross-sectional survey examined anatomy instructional modalities, narrative-assessment practices, reviewer structures, feedback characteristics, and faculty perceptions among respondents affiliated with LCME-accredited U.S. medical schools. Faculty from 155 institutions were invited; 58 responses were included in the analytic sample. Among 49 respondents who completed the instructional-modality item, cadaveric dissection was the most common instructional modality (*n* = 30), followed by prosection (*n* = 12), other approaches (*n* = 5), and virtual anatomy (*n* = 2). Nine respondents did not answer this item. Narrative assessment was reported by 31 of 58 respondents (53%). Because of small and uneven modality subgroup sizes, instructional-modality comparisons were interpreted descriptively. Among respondents reporting narrative assessment, feedback was typically delivered during the session or within 48 h, and most described their feedback as personalized. Respondents generally rated narrative assessment as beneficial. Reviewer workload was not associated with perceived benefit. Open-text responses highlighted barriers including time constraints, competing instructional demands, difficulty observing individual performance, and uncertainty regarding accreditation expectations. Narrative assessment was valued but implemented inconsistently among respondents, with workload and class size frequently identified as practical constraints. These findings describe current respondent-reported practices and identify practical considerations relevant to developing sustainable feedback strategies that uphold LCME standards in anatomy laboratories.

## Introduction

Narrative assessment has become a cornerstone of competency-based medical education by providing qualitative, descriptive information about learner behaviors like professionalism, teamwork and attitudes that may not be represented by traditional testing [1–3]. The Liaison Committee on Medical Education (LCME) is the accrediting body for medical education programs leading to the MD degree in the United States and Canada [4]. The LCME sets national standards that shape curriculum design and assessment expectations. The LCME Element 9.5 requires that a narrative assessment of a student’s performance be included in each required course and clerkship whenever teacher-student interaction permits this form of assessment. As a result, narrative assessment is not optional. However, how this requirement is interpreted and implemented in anatomy laboratories remains unclear. Consequently, the use of narrative assessment varies widely across programs and educational settings [5].

Recent efforts in medical education emphasize that early implementation of high-quality narrative assessment is pivotal for clinical preparedness by easing the transition to clinical training and fostering professional development [3–6]. Effective narrative assessment is generally timely, specific, individualized, and oriented towards learner improvement [1, 3, 7–9]. These characteristics overlap with principles described in feedback-as-coaching models, particularly the emphasis on timely, individualized, and improvement-oriented feedback [10–12]. Prior studies have noted that narrative assessment can vary in specificity and actionability [3, 6]. Accordingly, selected principles from feedback-as-coaching models were used as a conceptual lens to interpret reported feedback practices in this study, particularly regarding timeliness and personalization.

Anatomy laboratories present distinctive challenges for narrative assessment. In cadaveric dissection, students typically work in teams while faculty and teaching assistants circulate among multiple tables, resulting in brief and fragmented faculty-student interactions [4]. Although these encounters provide opportunities to observe teamwork, preparation, communication, and participation, they may limit direct individual observation and make it difficult to attribute performance to a single student [5, 13]. These challenges are particularly important because individualized feedback has been shown to be more effective than group-based feedback in promoting professional attitudes and behaviors in medical students during gross anatomy instruction [7, 8]. Furthermore, high student-to-faculty ratios reduce opportunities for sustained observation, while personal protective equipment and the dynamic laboratory environment can impede communication and make individualized feedback difficult [13–15]. Because effective narrative assessment depends on meaningful observation and individualized feedback, these factors may diminish the educational value of the feedback provided [1, 13–15].

In prosection-based or virtual environments, learners may participate through observation or discussion, or guided exploration rather than performing cadaveric dissection. While these approaches effectively support anatomical learning, they may provide fewer opportunities for faculty to directly observe procedural teamwork, task delegation, and other collaborative behaviors that emerge during hands-on dissection [16]. In contrast, if instructors are not simultaneously assisting with technical dissection tasks, they may have greater bandwidth to interact with students and form clearer impressions of individual learners. Differences among instructional modalities may therefore influence the feasibility, content, and perceived value of narrative assessment.

Existing literature has examined narrative assessment, feedback quality, assessor practices, and coaching across medical education [17]. However, less is known about how narrative assessment is implemented specifically within anatomy laboratories or whether practices differ according to the primary instructional modality. This study therefore describes current practices reported by anatomy educators affiliated with LCME-accredited U.S. medical schools, examines reported feedback characteristics and reviewer structures, compares practices descriptively across instructional modalities, and explores whether student-to-reviewer ratios and estimated narrative-assessment burden are associated with faculty perceptions of benefit.

## Methods

### Study Design

The protocol for this study was reviewed and given exempt status by the Institutional Review Board at the University of South Carolina (Pro00139704**).** The target population was anatomy educators involved in courses at LCME-accredited U.S medical schools.

### Survey Development

This study utilized a descriptive, cross-sectional survey to examine respondent-reported approaches to narrative assessment within anatomy laboratory courses at LCME-accredited U.S. medical schools. The study team consisted of anatomy faculty and medical education researchers involved in curriculum evaluation and assessment. Team members contributed to item development, survey refinement, and content validation. The survey examined current practices and explored whether student-to-instructor ratios were associated with perceived usefulness of narrative assessment. Descriptively, the study explored current practices relevant to meeting LCME Element 9.5’s narrative assessment requirements. The hypothesis-driven portion examined whether larger student-to-instructor ratios negatively affect perceived usefulness of narrative assessment in anatomy lab settings.

### Survey Content

The instrument consisted of 18 questions using multiple response formats, including Likert-scale, sliding-scale, ranked-choice, multiple-choice, select-all-that-apply, and open-ended items.

Survey development occurred through a three-stage iterative process. The initial draft was developed by the study team and subsequently revised into a second draft. The second draft underwent review by anatomy-adjacent faculty to assess relevance and clarity, followed by final revision and content validation by anatomy faculty.

Several survey domains were informed by prior national surveys of anatomical sciences education [4, 10, 11, 18]. Items were not adapted verbatim; rather, conceptual areas such as instructional modality, faculty participation, laboratory structure and assessment practices guided the development of similar domains in the present instrument. Additional domains were created to address narrative assessment practices specifically. Narrative assessment in anatomy laboratories primarily captures professional behaviors such as teamwork, preparation, communication, and engagement. These are elements not measured by written exams or practical identification tests. The survey focused on these observable behaviors rather than technical dissection skills or anatomical identification accuracy. All items underwent content validation by anatomy faculty. Edits were made based on feedback from colleagues and current trends in medical education. Survey items addressed multiple aspects of anatomy course structure, including classroom and laboratory hours, whether anatomy laboratories were scheduled as large- versus small-group activities, resources included in laboratory experiences, range of faculty participation, inclusion of peers as teachers, and methods of student assessment. Ranked‑choice items were used to characterize respondents’ primary instructional modality (cadaveric dissection, prosection, virtual anatomy, or other). Likert-scale items measured faculty-perceived benefit and faculty-reported student benefit. Separate Likert-scale items asked faculty to rate (1) their own perceived benefit of narrative assessment and (2) their perception of student benefit. Student‑to‑instructor ratios and workload scores were compared across institutions providing and not providing narrative assessment, and across reviewer‑identity groups. The survey was administered electronically using Qualtrics. Respondents could skip any question; therefore, item-level denominators vary, and missing responses were not imputed.

### Handling of Repeat Responses

Because the survey was intentionally designed to avoid collecting identifiable demographic information, including institution names, IP addresses, or other direct identifiers, it was not possible to definitively determine whether multiple responses originated from the same institution. Responses were screened for internal inconsistencies and conflicting course descriptions. Six responses containing conflicting or incomplete information were excluded from the analytic dataset. All remaining responses were treated as independent respondent-level entries, consistent with the study’s IRB-approved design and privacy protections. Accordingly, the number of unique institutions represented by the analytic sample could not be verified.

### Recruitment and Participants

All 155 LCME-accredited U.S. medical schools were eligible to participate. A list of eligible schools was obtained from the Liaison Committee on Medical Education website [19]. Institutional websites were then reviewed to identify a contact associated with each school. One direct email invitation was sent to a contact at each of the 155 eligible schools (155 emails total). When anatomy faculty contact information was publicly available, the invitation was sent directly to that faculty member; otherwise, it was sent to an available departmental or faculty-directory contact with a request that it be forwarded to the appropriate anatomy faculty. To broaden recruitment, the survey invitation was subsequently distributed through the American Association of Clinical Anatomists (AACA) listservs and MedEd listservs. Because listserv membership and institutional identifiers were not collected, the total number of individuals or institutions with access to the survey through the listservs could not be determined. Participation was voluntary, and no demographic information was collected to maintain faculty and institutional privacy.

### Data Analysis

#### Quantitative Analysis

Descriptive statistics were used to summarize respondent-reported practices. Missing item responses were not imputed; denominators reflect the number of respondents who answered each item. Because the virtual anatomy (*n* = 2) and other (*n* = 5) primary-modality groups were small and uneven, comparisons across instructional modalities were treated descriptively rather than inferentially. A paired t-test compared faculty-perceived benefit with faculty-reported student benefit among paired responses. Pearson correlation coefficients and simple linear regression were used to examine associations of reviewer workload and student-to-reviewer ratios with perceived-benefit scores. Statistical significance was defined as *p* < 0.05. A “workload score” was calculated by multiplying the number of narrative assessments delivered per academic year by the number of students per reviewer. The workload score was designed as a composite measure reflecting both the number of narrative assessments delivered and the number of students assigned to each reviewer. This measure aligns with the study’s aim of exploring whether reviewer burden relates to perceived benefit. This measure reflects only the observable components captured by the survey and does not account for additional faculty responsibilities or depth of feedback.

Reviewer identity was categorized based on who provided narrative assessment for each response. Respondents reported using faculty reviewers, TAs (teaching assistants), peer evaluation, self-reflection, or mixed reviewer groups. For workload calculations, only faculty and teaching assistants who indicated participating in narrative assessments were included as reviewers. Peer evaluation and self-reflection were excluded from workload score calculations, as these activities represent different levels of responsibility, but may still contribute to some faculty reviewer burden if faculty are responsible for compiling or reviewing peer and self-assessments.

Faculty‑perceived benefit refers to faculty members’ own views of the usefulness of narrative assessment. Faculty‑reported student benefit refers to faculty perceptions of how beneficial students find narrative assessment. No direct student data was collected.

#### Qualitative Analysis

Qualitative free-text responses were grouped thematically to identify common challenges, reported practices, and perceived barriers. Free‑text responses were coded by a single reviewer using an inductive thematic approach. Repeated concepts across responses were identified and documented as themes. Due to the small number of qualitative entries, team‑based coding and inter‑rater reliability were not feasible.

## Results

### Responses and Survey Participation

During 2022–2023, direct email invitations were sent to an anatomy faculty member or department at each of 155 different LCME-accredited U.S. medical schools. Of the 64 returned surveys, 21 originated from direct email, 24 through the MedEd listserv, and 19 through the AACA listserv. Six responses were excluded because of conflicting or incomplete information, yielding a final analytic sample of 58 responses. Because institutional identifiers were not collected and listserv distribution broadened access to the survey, the number of unique institutions represented by these responses could not be determined. Not every respondent answered every survey item; item-specific denominators are therefore reported where relevant.

### Instructional Modality Distribution

The primary instructional modality was collected using a ranked-choice question. Of the 58 respondents in the analytic sample, 49 completed this item, and nine left it blank. Cadaveric dissection was ranked as the primary instructional modality by 30 respondents (*n* = 30), followed by prosection (*n* = 12), other approaches (*n* = 5), and virtual anatomy (*n* = 2) (Table 1). Because the virtual anatomy subgroup contained only two respondents and the other category represented heterogeneous approaches, inferential comparisons across instructional modalities were not performed; modality findings are reported descriptively.

**Table 1:** Ranked instructional tools used in anatomy

Instructional Tool	Rank 1	Rank 2	Rank 3	Rank 4	Notes / Examples from “Other” Category
Cadaver	30	17	5	1	–
Prosection	12	26	12	3	–
Virtual Anatomy Tools	2	5	27	18	Includes various digital platforms
Other (combined)	5	1	7	25	Subthemes listed below
• Models	2	–	3	1	Physical anatomic models
• Plastinates	–	–	3	1	Preserved anatomical specimens
• Radiology / Radiographs	1	–	2	1	Use of imaging modalities
• Learning Management Systems	–	–	1	–	LMS-based anatomy activities
• Case-Based Learning	–	–	–	1	Team-based or scenario-based sessions

### Narrative Assessment Practices

Among all 58 respondents, 31 (53%) reported providing narrative assessment in the anatomy laboratory, while 27 (47%) reported no narrative assessment in this setting. Student-to-instructor ratios were calculated by dividing the number of students in the class by the total number of faculty and TAs (teaching assistants) reported in the anatomy laboratory and were summarized descriptively according to narrative-assessment status.

**Fig. 1:**
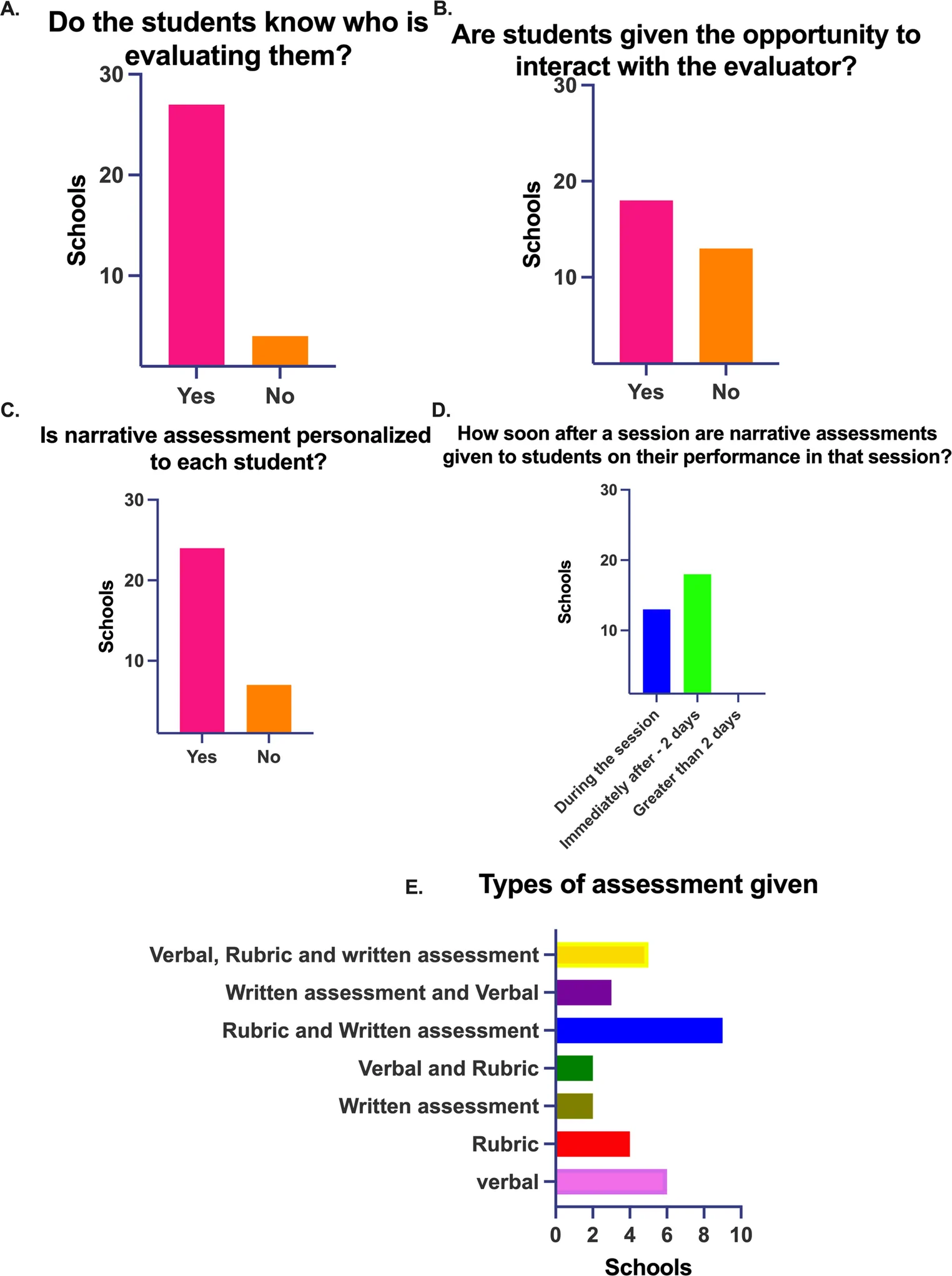
Narrative assessment landscape across responding medical institutions. Institutional practices regarding narrative assessment including (**A**.) whether students know who is evaluating them. (**B**.) Whether students have opportunities to interact (Respond to) their evaluator. (**C**.) Whether narrative assessment are personalized. (**D**.) The timing by which narrative assessments are delivered. (**E**.) The types of feedback modalities used

The 31 respondents who reported narrative assessment described a wide variation in assessment modality, structure, and delivery. Key components of the narrative assessment landscape are reported in Fig. 1. Most respondents indicated that students knew who was evaluating them (27 yes vs. 4 no), while fewer reported that students had opportunities to interact directly with their evaluator (18 yes vs. 13 no). Most reported that narrative assessment was personalized to each student (24 yes vs. 7 no). All respondents reported providing feedback either during the evaluated session or within two days (13 during the session vs. 18 within two days). Feedback modalities also varied. Five respondents (16%) used verbal, rubric-based, and written assessments; three respondents (10%) used written and verbal assessments; nine respondents (29%) used rubric-based and written assessments; two respondents (6%) used verbal and rubric-based assessments; two respondents (6%) used solely written assessments; four respondents (13%) used solely rubric-based assessments; and six respondents (19%) used solely verbal assessments. These categories account for all 31 respondents, with percentages differing slightly from 100% because of rounding.

### Reviewer Structures

**Fig. 2:**
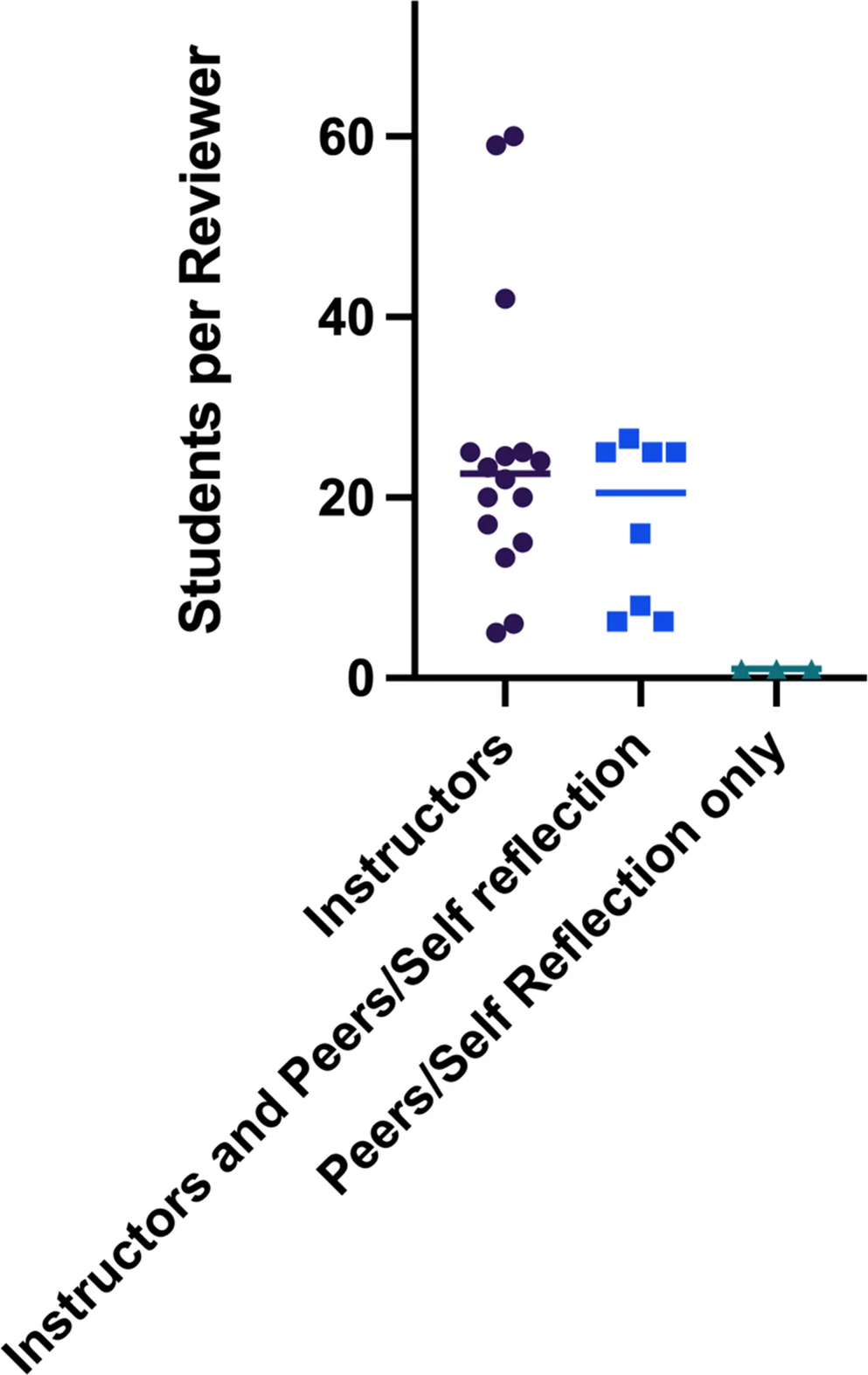
Students per reviewer by reviewer type. The distribution of students per reviewer across three reviewer structures: Instructors only, instructors combined with peer and self-reflection, and peer/self-reflections only. Institutions reporting only peer/self-reflection are shown as a 1-1 ratio

Respondents reported diverse structures of anatomy lab staffing. Student-to-instructor ratios varied widely across institutions, and reviewer identity also differed: Some respondents reported relying only on faculty to provide narrative assessment, while others reported using TAs, peer evaluation/self-reflection, or mixed reviewer groups (Fig. 2). While instructors were defined above as faculty and TAs in the anatomy lab, reviewers are here defined as the instructors involved in providing narrative assessment to students. Only three respondents relied on peer evaluation or self-reflection and were therefore categorized together due to the low number of responses. Respondents reporting peer evaluation and self-reflection reporting institutions showed a student-to-reviewer ratio of 1:1. Responses in which instructors alone were responsible for reviewing students showed the widest range of student-to-reviewer ratios, including several institutions with more than 40 students per reviewer. Responses using a combination of instructors and peers/self-reflection demonstrated more moderate and clustered ratios, with most values between 10 and 25 students per reviewer.

### Benefit Associations

**Fig. 3:**
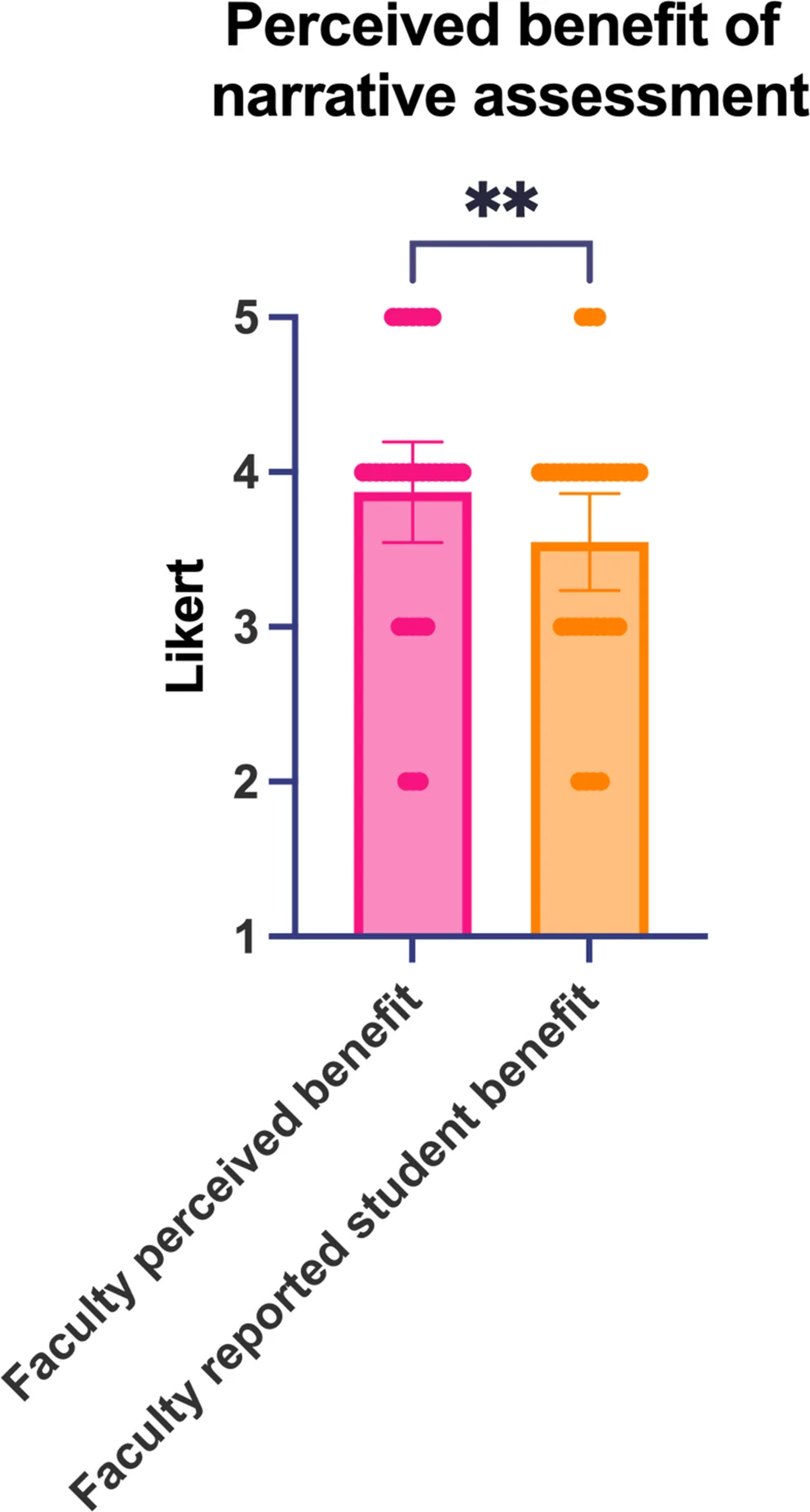
Likert perceived benefit. Stacked likert distributions with paired t-test showing how beneficial faculty perceive narrative assessment to be for themselves, and for students. Ratings range from 1 (Extremely unbeneficial) to 5(Extremely beneficial), with most institutions selecting categories 4 and 5. Different between groups: *P*=0.0099

Faculty perceptions of the benefit of narrative assessment and faculty-reported perceptions of student benefit were collected using Likert scales (Fig. 3). Most respondents rated narrative assessment as “somewhat beneficial or extremely beneficial” for both faculty and students. Very few respondents reported narrative assessment as unbeneficial. Faculty-perceived benefit scores were higher than faculty-reported student benefit scores, although both distributions clustered in the upper half of the 5-point scale (paired t-test, *p* = 0.0099).

Student-to-reviewer ratio was not significantly associated with either faculty-perceived benefit or faculty-reported student benefit; regression models explained less than 2% of the variance in these benefit ratings (R²<0.02). Reviewer workload was likewise not significantly associated with perceived benefit. Faculty-perceived benefit showed a weak, non-significant correlation with workload (*r* = 0.159, *p* = 0.411; R²=0.025), while faculty-reported student benefit showed a slightly stronger but still non-significant correlation (*r* = 0.245, *p* = 0.200; R²=0.060). Because several feedback-modality combinations contained small numbers of respondents, benefit patterns across feedback modalities were treated descriptively, and no inferential comparison was retained.

### Timeliness and Personalization of Narrative Assessment

Most respondents providing narrative assessment reported that feedback was personalized (24 of 31 institutions), whereas 7 institutions reported delivering non-personalized or template-based feedback.

Narrative assessment was delivered either during the session or within 48 h: 13 respondents reported delivery during the session and 18 within 48 h. No respondents reported delivery later than 2 days after the session. Benefit ratings appeared similar descriptively across personalization and timing groups; no inferential comparisons were retained because of the small group sizes.

### Qualitative Themes

Eighteen respondents answered the open-ended question, ”Are *there any other comments about narrative assessment at your institution that you would like to provide?*” Recurrent themes described the benefits and challenges of utilizing narrative assessment in anatomy laboratories. The most common theme (*n* = 9) involved workload limitations. Examples of workload limitations are provided below:

“Faculty does not have enough time to interact with students to provide meaningful feedback all the time.”

“The time concerns and crunch to address student didactic questions in lab preclude their adoption at this time.”

“Faculty feels generally overwhelmed by the additional work to provide the assessment.”

The second theme (*n* = 5) reflected the value of narrative assessment when implemented well:

“Provided for student benefit. Does not contribute to course score or grade.”

“…Could potentially be a good tool….”

“Sadly we do not conduct narrative assessments in the anatomy lab. While they would be beneficial, the time concerns and crunch to address student didactic questions in lab.”

Three respondents described the use of peer evaluation or self-feedback as an approach to narrative assessments:

“We have students rate each other, and themselves… and then use this feedback to construct summary comments from faculty/TAs.”

“Students give feedback to the peers they work with at the dissection table and also complete a self-reflection.”

Another three responses mentioned expressed confusion regarding LCME expectations:

“I’ve been involved with anatomy teaching for 30 years and this is the first time I’ve heard of narrative assessment.”

“We give verbal assessment in lab but I’ve never done narrative assessment, as described in this survey.”

Two respondents noted difficulty observing individual students in anatomy lab (*n* = 2):

“Most of the time it is hard to make observations about who is doing what, or hear conversations between team members.”

“I think it would be very difficult to provide narrative assessment… I don’t know what I would provide narrative assessment on.”

For the survey item, *“Are there any measures of success or follow-up to see if narrative assessment was beneficial to students at a later date?”* three respondents reported asking students about narrative assessment value on end-of-course evaluations, one reported that their institution had published its narrative assessment practices, and 14 responded “no.”

## Discussion

This study describes how respondents affiliated with LCME-accredited medical schools reported implementing narrative assessment within anatomy laboratories. Of the 58 analytic responses, 31 reported implementing narrative assessment, yet approaches varied considerably with respect to delivery modality, reviewer structure, evaluator interaction, and personalization. Despite variation in reported practices, faculty generally perceived narrative assessment as beneficial, while qualitative responses consistently emphasized barriers such as time constraints, competing instructional responsibilities, limitations in individual observation, and uncertainty surrounding accreditation expectations. Importantly, neither student-to-reviewer ratios nor the exploratory reviewer workload measure demonstrated significant associations with faculty-reported perceptions of benefit.

The finding that only 31 of 58 respondents reported narrative assessment in the anatomy laboratory should not be interpreted as indicating that the remaining respondents represented institutions out of compliance with LMCE Element 9.5. The standard requires narrative descriptions of student performance whenever teacher-student interaction permits but does not specify that these assessments must occur in the anatomy laboratory [20, 21]. Therefore, anatomy laboratories may only represent one of several educational settings in which a curriculum addresses this requirement, including longitudinal skills courses, small-group learning, or other preclerkship activities. In addition, because institutional identifiers were not collected, the number of unique medical schools represented by the respondent sample could not be verified. These results are therefore best interpreted as respondent-reported anatomy laboratory practices rather than an institution-level estimate of compliance or implementation.

The anatomy laboratory presents unique challenges in affording individualized narrative assessment. In cadaveric dissection, faculty and TAs supervise multiple student teams simultaneously, in addition to technical instruction, laboratory safety, and addressing student questions [4]. These competing responsibilities may limit opportunities for adequate observation of learners on an individual level despite frequent faculty presence [5, 13]. Conversely, prosection-based or virtual anatomy curricula may afford more focused interaction with individual students, while potentially reducing opportunities to observe certain behaviors such as teamwork, procedural participation, and task delegation. Future studies with larger instructional-modality subgroups are needed to determine whether modality influences feedback practices; the education context itself may contribute to the diverse approaches reported by respondents.

Contrary to our hypothesis, neither student-to-reviewer ratios nor the exploratory reviewer workload measure demonstrated significant associations with faculty perceptions of narrative assessment benefit. These findings should be interpreted with caution. The workload estimates in this study were simplified and reflected only narrative assessment volume and reviewer assignment. They did not account for competing educational responsibilities, time required to formulate meaningful feedback, administrative obligations, or differences in feedback depth and quality. The absence of statistical associations should not be interpreted as evidence that workload does not influence narrative assessment. Rather, it suggests that the variables measured in this study did not adequately explain variation in perceived benefit.

Interestingly, the qualitative findings complemented rather than contradicted the quantitative analysis. Although measured workload variables were not associated with perceived benefit, respondents repeatedly endorsed insufficient time, completing instructional demands, and difficulty observing students as barriers to implementation. Faculty also expressed uncertainty regarding what defines adequate narrative assessment, as well as ambiguity about whether anatomy labs are expected to contribute to LCME Element 9.5. These findings suggest that anatomy educators recognize the value in narrative assessment but acknowledge practical obstacles that limit consistent implementation.

Among respondents providing narrative assessment, nearly all respondents reported delivering feedback during laboratory sessions or within 48 h, and most described their feedback as being individualized. These characteristics are consistent with recommendations from the broader medical education literature, which emphasizes that effective feedback is timely, specific, and clearly connected to the observed learner’s performance [1, 3, 7–9, 22]. However, this study evaluated only structural characteristics of reported feedback practices. It did not assess the content, specificity, or actionability of narrative comments. Consequently, these findings should not be interpreted as evidence that reported feedback consistently met established standards for high-quality narrative assessment.

Feedback-as-Coaching provides one useful framework through which these findings may be interpreted. Coaching models emphasize longitudinal learner development, individualized guidance, learner engagement, and ongoing dialogue rather than isolated evaluative encounters [7, 10–12, 21, 23, 24]. Of the responding faculty surveyed, many align with these principles. However, this survey did not determine whether participating respondents’ programs intentionally used coaching models as their narrative framework. In addition, this study did not compare coaching-based approaches with alternative feedback strategies. Accordingly, Feedback-as-Coaching should be viewed as one potential interpretive lens rather than a conclusion supported directly by the present data.

### Study Implications

The findings have practical implications for anatomy educators seeking to strengthen their formative assessments. Rather than suggesting that effective narrative assessment requires lower student-to-faculty ratios alone, the results indicate that successful implementation may rely on clearly defining observable learning behaviors, assigning responsibility for assessment, and developing a feasible process that integrates naturally into existing laboratory workflows. Observable behaviors such as preparation, teamwork, communication, professionalism, and engagement may provide measurable targets for narrative assessments within anatomy laboratories without replacing traditional measures of knowledge or performance.

### Study Limitations

This study has several limitations. A conventional response rate could not be calculated because the survey was distributed through listservs for which membership and institutional reach were unknown. Although 155 direct invitations were sent, institutional identifiers were not collected; therefore, the number of unique institutions represented and the possibility of more than one response from the same institution could not be determined. Participation was voluntary, introducing the potential for response bias. Respondents could skip survey items, resulting in variable denominators across analyses. Student perceptions were not collected directly but were interpreted through faculty-reported student benefit; therefore, true student experience may not be fully represented. The survey assessed structural characteristics of feedback but did not evaluate the quality, specificity, or educational impact of narrative comments. The workload measure represented an exploratory estimate of reviewer burden and by itself cannot capture the full complexity of faculty obligations. The small virtual subgroup (*n* = 2) and heterogeneous other-modality group precluded meaningful inferential comparisons across instructional modality. Finally, the limited number of open-ended responses required descriptive qualitative analysis by a single reviewer, reducing the depth of qualitative interpretation.

### Future Directions

Future research should examine narrative assessment from the learner perspective, evaluate the quality and actionability of narrative comments, and investigate whether instructional modality influences the feasibility and educational impact of narrative assessment. Longitudinal studies examining professionalism, teamwork, feedback literacy, and subsequent clinical performance would further elucidate how anatomy-based narrative assessment contributes to medical students’ development throughout medical training.

In conclusion, narrative assessment was reported by approximately half of respondents and was incorporated through diverse reviewer structures and feedback practices. Although faculty largely perceived narrative assessment to be beneficial, practical challenges such as observation time, competing instructional responsibilities, and uncertainty regarding implementation remain common. These findings provide a national description of respondent-reported practices among responding institutions and identify opportunities for consistent and feasible approaches to narrative assessment within anatomy laboratories.

## Data Availability

Data is available upon request and at the discretion of the authors.
